# 

**Published:** 1981

**Authors:** 


JULY/OCTOBER 1981
VOLUME 96 (iii/iv)
NUMBER 359/360
Published Quarterly
Annual Subscription £6 Post Free

### BRISTOL MEDICO-CHIRURGICAL JOURNAL

### Contents

3 **The Postmortem is not Dead**

N. J. Brown

*Presidential address to Bristol Medico-Chirurgical Society, 8th October 1980*

14 **The Surprises of General Practice**
Philip Radford

17 **Skull X-Ray and CT. Scan in the Management of Head Injuries**
D. J. Tawn

20 **South West Surgeons Club**
Abstracts of papers given at the meeting in Cork, 1st-2nd May 1982

25 **Book Review**
Operative Surgery and Management

26 **Examination Results**

Established 1883

### BRISTOL MEDICO-CHIRURGICAL JOURNAL

Incorporating the Medical Journal of the South West

### Editorial Committee

EDITOR
*Mr. M. G. Wilson*
*Litfield House, Clifton, Bristol, BS8 3LS*
ASSISTANT EDITOR
*Dr. G. R. Tribe*
*Southmead Hospital, Bristol, BS10 5NB*
MEMBERS
*Dr. Rhys Davies*
*Dr. M. B. Lennard*
*Dr. D. W. Wright*
*The Hon. Treasurer of the Society*
*The Hon. Secretary of the Society*
SECRETARY
*Mr. B. P. Jones, The Library, Medical School,*
*University Walk, Bristol, BS8 1TD*

### Bristol Medico-Chirurgical Society

PRESIDENT 1980/81
*Dr. N. J. Brown*
PRESIDENT ELECT
*Dr. R. P. Warin*
HONORARY SECRETARY
*Dr. H. M. Norman*
*Hillside, Vicarage Lane, Barrow Guerney*
HONORARY TREASURER
*Dr. J. Penry*
*Southmead Hospital, Bristol, BS10 5NB*

The Society was founded in 1874. In 1883 publication of the Journal began and has continued quarterly ever since then. During the years 1949 to 1962 it appeared under the title of 'The Medical Journal of the South West'.

### Notice to Contributors

*The Journal is especially concerned to provide a place for the recording of medical thought, interests, and practice in and around Bristol. It therefore welcomes articles that, as well as being of immediate interest to members, will document the local and contemporary medical scene for our successors.*

*Original articles are invited provided that they have not been published elsewhere. Illustrations should be supplied ready for reproduction. Line drawings should be in Indian ink on firm white paper or board. The author will be informed if it is found necessary to ask him to pay for the printing of photographs, etc.*

*The following contributions will be especially welcome: notes of forthcoming events, meetings held, degrees conferred, staff appointments, honours and public appointments and other local news.*

*Advice on preparation can be obtained from the Editor.*

Printed by the University of Bristol Printing Unit

### University of Bristol Faculty of Medicine

### THE DEGREE OF DOCTOR OF PHILOSOPHY
**June 1981**
**Dissertations Approved**

Peter John MILLER
Rosemary Jane READ
John William SEAR

### THE DEGREE OF MASTER OF SCIENCE IN MEDICAL SCIENCES
**June 1981**
**Dissertation Approved**

Michael Hugh HINTON

### THE DEGREE OF DOCTOR OF MEDICINE
**June 1981**
**Dissertation Approved**

Richard PAISEY

### FINAL EXAMINATION FOR THE DEGREES OF M.B., Ch.B.
**June 1981**

**WITH HONOURS**
Shona Ellen IRVING
  with Distinction in Community Health
Barry Leonard PIZER
  with Distinctions in Child Health and in Obstetrics & Gynaecology
Shenaz RAMTOOLA
  with Distinction in Mental Health
Claire Amanda SAMUEL
  with Distinctions in Medicine, in Surgery and in Pathology
Christopher John UPTON
  with Distinction in Mental Health
Peter John WILLIAMSON
  with Distinctions in Child Health and in Pathology

**PASS**
Judyth Elizabeth AARONS
Janet Elizabeth ADAMS
Alison Wendy ANNISS
  with Distinction in Mental Health
Toqeer ASLAM
Julie Anita BAKER
Tamara Mary BARLOW
Simon Jocelyn BARRINGTON-WARD
Gillian BEDFORD
Mitradev BOOLELL
Maria-Teresa BREDOW
David Heron BREWSTER
Judith Denise BROOKS
Graham St.John BROWNLIE
  with Distinctions in Obstetrics & Gynaecology and in Community Health
Sarah Kate BRUML
James Richard BUCKLE
Diane Louise Empain BULLOCK
Timothy Stuart BURGE
Juliet Mary CAMPLING
Pun Hon CHANG
Patricia Mary CHARLETON
Mark Jonathon Dominic COBBY
Rosalind Anne DAINTREE
Rosemary Jean DALTON
Philip Ashoke DAS
Michael John DAVIES
Malini Jayant DESAI
Clare Louise DETSIOS
Colin Alan DONALDSON
John Cornelius DOYLE
Elizabeth Carolyn ELTON
Catherine Mary Lodwick EVANS
John Anthony Lodwick EVANS
Adrian Jude FARRELL
Rowena Diana FIELDHOUSE
Stephanie Noelle FOOT
Ian Robert GILCHRIST
Alison Rosemary GILLAMS
Brian George GREEN
Philip Antony Michael GUISE
Christine Asya HAFFNER
Moira Anne HAMILTON
Ginette Lesley HARRISON
William James HINTON
Kirsten Isabel HOPKINS
David Stanley HUGHES
Anne Judith HUMPHREYS
Paul HUNT
Simon Lindsay HUNTER
Wanda Irena ISKRZYNSKA
Judith Anne Marjorie JACKSON
Jane Elizabeth JONES
Lesley Margaret JORDAN
Damian KENNY
Linda Ruth KEYLOCK
Paul Richard KINNERSLEY
Barbara Louise KIRKPATRICK
Kim Charles KRARUP

Heather Joan LAMBERT
John Francis LANCASTER
Clare Elizabeth LAXTON
John LEIGH
John Owain LLEWELLYN
Christine Cecilia LOCK
Simon Wyndham LOWE
Rhona Isabel MACPHERSON
Adam Paul MASTERS
David Gibson MCGAUGHEY
Janine Elizabeth MENDHAM
Sally Anne MORAN
Martin Hill MORSE
Jeffrey Peter Stanley PARROTT
Ian Stuart PATTERSON
Andrew Nicholas PATON
Elizabeth Sara POTTER
Michael John POTTS
Richard Maguire REDMOND
John Keith ROBERTSHAW
Philip James ROBINSON
Jane Frances SCHULTE
Frances Ruth SELF
David SEMERARO
Jean Maria SHERRINGTON
Jane Fiona SMILLIE
Katherine Sarah Howard SMITH
James Peter SOUTHERN
Elizabeth Jane Rosann STEWART
William Paul STROSS
with Distinction in Surgery
Timothy Martin TAYLER
Alistair John TAYLOR
Wendy Jane TAYLOR
Alan Donald THOMAS
Lindsey Grace Clarke THOMPSON
Ann Elizabeth TONGE
Helen Denise TOWNER
Christopher TOWNSEND
Susan Mary UNDERWOOD
Mary Louise VOGEL
David Gerrard WALBRIDGE
Christopher John WILKINS
Catherine WOODWARK
Gillian Elizabeth WORSLEY

